# Twisted Ways to Find Plane Structures in Simple Drawings of Complete Graphs

**DOI:** 10.1007/s00454-023-00610-0

**Published:** 2024-01-03

**Authors:** Oswin Aichholzer, Alfredo García, Javier Tejel, Birgit Vogtenhuber, Alexandra Weinberger

**Affiliations:** 1 Institute of Software Technology, Graz University of Technology, Graz, Austria; 2 Departamento de Métodos Estadísticos and IUMA, Universidad de Zaragoza, Zaragoza, Spain

**Keywords:** Simple drawings, Simple topological graphs, Disjoint edges, Plane matching, Plane path, 05C10, 05C38, 05C62

## Abstract

Simple drawings are drawings of graphs in which the edges are Jordan arcs and each pair of edges share at most one point (a proper crossing or a common endpoint). A simple drawing is c-monotone if there is a point *O* such that each ray emanating from *O* crosses each edge of the drawing at most once. We introduce a special kind of c-monotone drawings that we call generalized twisted drawings. A c-monotone drawing is generalized twisted if there is a ray emanating from *O* that crosses all the edges of the drawing. Via this class of drawings, we show that every simple drawing of the complete graph with *n* vertices contains $\Omega (n^{\frac{1}{2}})$ pairwise disjoint edges and a plane cycle (and hence path) of length $\Omega (\frac{\log n }{\log \log n})$. Both results improve over best previously published lower bounds. On the way we show several structural results and properties of generalized twisted and c-monotone drawings, some of which we believe to be of independent interest. For example, we show that a drawing *D* is c-monotone if there exists a point *O* such that no edge of *D* is crossed more than once by any ray that emanates from *O* and passes through a vertex of *D*.

## Introduction

In this work we investigate simple drawings of complete graphs, in particular plane subdrawings in them and special classes of them. *Simple drawings* are drawings of graphs in the plane or on the sphere such that the vertices are distinct points in the plane, the edges are Jordan arcs connecting their endpoints, and every pair of edges intersects at most once either in a proper crossing (of exactly two edges) or in a shared endpoint. We say that a drawing or subdrawing is *plane* if it contains no crossings. A graph is *planar* if there exists a drawing of the graph which is plane.

Two simple drawings on the sphere are called *strongly isomorphic* if there is a homeomorphism of the sphere that maps one drawing into the other. Similarly, two simple drawings in the plane are called strongly isomorphic if their homeomorphic drawings on the sphere are strongly isomorphic. (These homeomorphic drawings on the sphere exist by the general Jordan–Schoenflies theorem [11, 25].) Two simple drawings *D* and $D'$ (in the plane or on the sphere) are *weakly isomorphic* if there is a bijection between the vertices of *D* and $D'$ such that adjacency is preserved and any pair of edges in *D* crosses exactly when the corresponding pair of edges in $D'$ crosses. In this paper, we are mostly working with simple drawings in the plane, though everything holds equivalently also on the sphere. We often consider these drawings up to (strong or weak) isomorphism, where the type of isomorphism depends on the context.

In the past decades, there has been significant interest in simple drawings. Questions about plane subdrawings of simple drawings of the complete graph on *n* vertices, $K_n$, have received particularly close attention. Rafla [31] conjectured that for any $n\ge 3$, every simple drawing of $K_n$ contains a plane Hamiltonian cycle. The conjecture has been shown to hold for $n \le 9$ [2], as well as for several special classes of simple drawings, like straight-line, x-monotone, and cylindrical drawings. But it still remains open in general. If Rafla’s conjecture is true, then this would immediately imply that every simple drawing of the complete graph contains a plane perfect matching (if the number of vertices is even) and a plane Hamiltonian path. However, to-date even the existence of such a matching or such a path is still unknown.

Note that the behavior of general simple drawings with respect to plane subdrawings is often very different from the one of special simple drawings like straight-line drawings. For example, in contrast to straight-line drawings, simple drawings of $K_n$ in general do not contain triangulations, that is, plane subdrawings where all faces (except at most one) are 3-cycles. Moreover, different edge-maximal plane subdrawings of a given simple drawing of $K_n$ might have different numbers of edges, and it is NP-complete to decide whether a given simple drawing of $K_n$ contains a plane subdrawing of size *k* [18].

There has been a considerable series of results successively improving the lower bound of the number of disjoint edges contained in any simple drawing of $K_n$. In 2003, Pach, Solymosi, and Tóth [28] showed that any simple drawing of $K_n$ contains $\Omega ((\log n )^{\frac{1}{6}})$ disjoint edges. This bound has been improved to $\Omega (\frac{\log n }{\log \log n})$ by Pach and Tóth [29] in 2005, to $\Omega ((\log n)^{1+\varepsilon })$ by Fox and Sudakov [14] in 2009, and to $\Omega (n^{\frac{1}{3}})$ by Suk in 2013, where the last bound has been reproved via different techniques by Fulek and Ruiz-Vargas in 2013 [17] and 2014 [15]. Finally, in 2017 Ruiz-Vargas [33] showed a bound of $\Omega (n^{\frac{1}{2}-\varepsilon })$ for any $\varepsilon >0$. We further improve this bound, showing that every simple drawing of $K_n$ contains $\Omega (n^{\frac{1}{2}})$ disjoint edges.^1^

### Theorem 1.1

Every simple drawing of $K_n$ contains at least $\lfloor \sqrt{\frac{n}{48}} \rfloor$ pairwise disjoint edges.

For connected plane substructures, it follows from the definition of simple drawings that every such drawing of $K_n$ contains *n* plane spanning stars (a star is a tree in which all edges are incident to the same vertex). General plane trees are harder to find. Pach, Solymosi, and Tóth [28] showed that any simple drawing of $K_n$ has a plane subdrawing strongly isomorphic to any fixed tree with $O((\log n )^{\frac{1}{6}})$ vertices. This implies that every such drawing contains a plane path of length $\Omega ((\log n )^{\frac{1}{6}})$. We improve this bound by showing a lower bound of $\Omega (\frac{\log n }{\log \log n})$ for the length of a plane cycle, which in turn implies the same bound also for plane paths. An extended abstract containing the result for paths has been previously presented at EGC 2021 [6]. To the best of our knowledge, no non-trivial lower bound on the size of plane cycles had been published before.

### Theorem 1.2

Every simple drawing of $K_n$ contains a plane cycle of length $\Omega (\frac{\log n }{\log \log n})$.

Very recently, Suk and Zeng [34] improved the number of vertices from the above-mentioned result on fixed trees [28] to $(\log n)^{\frac{1}{4} - o(1)}$. In that work, they independently also obtained the same bound for plane paths as we do (partially using similar statements but quite different proof techniques).

To prove Theorem 1.1 and Theorem 1.2, we work with special families of simple drawings. In particular, we use c-monotone drawings and subfamilies of them.

### Definition 1.3

A simple drawing *D* is **c-monotone** (short for circularly monotone) if there is a point *O* such that any ray emanating from *O* intersects any edge of *D* at most once.

We label the vertices of c-monotone drawings $v_1, \ldots , v_n$ in counterclockwise order around *O*. We remark that Fulek and Ruiz-Vargas [15, 17] also used c-monotone drawings^2^ to show their bound on the number of disjoint edges in any simple drawing of $K_n$.

The two subfamilies of c-monotone drawings we mainly work with are the well-known family of x-monotone drawings (see further below for a discussion); and the new family of generalized twisted drawings, which we have introduced because they are a main ingredient for proving Theoremd 1.1 and  1.2.

### Definition 1.4

A simple drawing *D* of $K_n$ is **generalized twisted** if there is a point *O* such that *D* is c-monotone with respect to *O* and there exists a ray *r* emanating from *O* that intersects every edge of *D*.

**Fig. 1:**
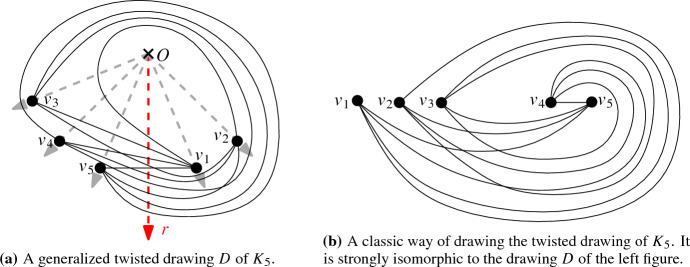
Two strongly isomorphic drawings of $K_5$ representing the twisted drawing

We label the vertices of generalized twisted drawings such that the ray *r* emerges from *O* between the ray to $v_1$ and the one to $v_n$ (and, like in all c-monotone drawings, $v_1, \ldots , v_n$ are labeled in counterclockwise order around *O*). Figure 1a shows an example of a generalized twisted drawing of $K_5$.

The name generalized twisted is derived from the known class of twisted drawings. A simple drawing of $K_n$ is *twisted* if there is a labeling of the vertices to $v_1, v_2, \ldots , v_n$ such that $v_iv_j$ ($i<j$) crosses $v_kv_l$ ($k<l$) if and only if $i<k<l<j$ or $k<i<j<l$. It is known that for any *n*, there exists a twisted drawing of $K_n$ [21]. Further, this drawing is unique up to weak isomorphism by definition. Generalized twisted drawings are a generalization of twisted drawings in the sense that one can show that every twisted drawing is strongly isomorphic to a generalized twisted drawing. In contrast, most generalized twisted drawings are not even weakly isomorphic to twisted drawings; see the discussion in the conclusion. Figure 1b shows a classic way of representing the (up to strong isomorphism unique) twisted drawing of $K_5$ [21]. It is strongly isomorphic to the generalized twisted drawing in Fig. 1a.

Originally introduced by Harborth and Mengersen in 1992 [21], twisted drawings have been thoroughly investigated; see for example [4, 9, 13, 24, 27, 28, 34]. Pach, Solymosi, and Tóth [28] showed that every simple drawing of $K_n$ contains an induced subdrawing of a complete graph on $\Omega (\log ^{\frac{1}{8}}n)$ vertices which is either *convex*^3^ or *twisted*. They used this to obtain their previously mentioned tree-existence result. Recently, Suk and Zeng [34] improved the lower bound on the size of a convex or twisted induced subdrawing of a complete graph in any simple drawing of $K_n$ to $(\log n)^{\frac{1}{4} - o(1)}$ vertices.

In this paper, we investigate generalized twisted drawings and use their rich structure to prove Theorems 1.1 and  1.2. As one of the most important properties for those proofs, we show the following.

### Theorem 1.5

Every generalized twisted drawing *D* of $K_n$ contains a plane Hamiltonian path. Moreover, if $n\ge 3$ is odd, then *D* also contains a plane Hamiltonian cycle.

Note that for any plane substructure in a simple drawing, the according substructure in any strongly or even just weakly isomorphic drawing is plane as well. Hence we could also state Theorem 5 starting with "Any simple drawing of $K_n$ that is weakly isomorphic to a generalized twisted drawing...". This holds analogously for several other statements throughout this work. For simplicity, we stick with the shorter (though at first glance more restricted) statements.

**Fig. 2:**
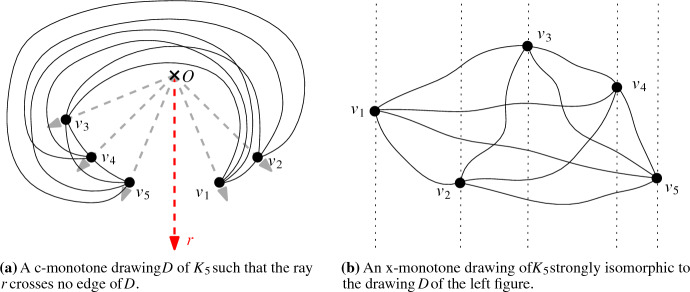
Two strongly isomorphic monotone drawings of $K_5$

To gain more insight into c-monotone drawings and to show Theorem 1.2, we further use x-monotone drawings. A simple drawing in the plane is *x-monotone* if any vertical line intersects any edge of the drawing at most once; see Fig. 2b for an illustration. This family of drawings has been studied extensively in the literature; see for example [3, 8, 10, 16, 30].

For a characterization of x-monotone drawings via c-monotone drawings, consider c-monotone drawings in which there exists a ray *r* from the origin *O* that does not cross any edge of the drawing. Any such drawing is strongly isomorphic to an x-monotone drawing. One way to obtain this isomorphic x-monotone drawing is the following: We transform the plane such that *O* is sent to infinity and all rays through *O* become (parallel) vertical rays, with *r* being the vertical line at infinity. (This process is an isotopy, so the resulting drawing is strongly isomorphic to the original one. One can also imagine the transformation as cutting the drawing open along *r* and then stretching it until it is x-monotone.) On the other hand, it is easy to see that every x-monotone drawing in the plane is strongly isomorphic to a c-monotone drawing (for example by using the inverse of the above described transformation).

Figure 2a shows a c-monotone drawing *D* of $K_5$ where no edge crosses the ray *r*, and Fig. 2b shows an x-monotone drawing of $K_5$ strongly isomorphic to *D*.

We call simple drawings that are strongly isomorphic to x-monotone drawings *monotone* drawings. In particular, any c-monotone drawing for which there exists a ray emanating from *O* that crosses no edge of the drawing is monotone. Note that generalized twisted drawings are somewhat opposite to such monotone drawings, in the sense that they contain a ray emanating from *O* that crosses every edge of the drawing. We explore further connections in the proof of Theorem 1.2 and show, amongst other things, that every c-monotone drawing of $K_n$ contains a drawing of $K_{\lceil \sqrt{n}\rceil }$ as an induced subdrawing that is either generalized twisted or x-monotone (Theorem 6.3).

We further study c-monotone drawings, especially in the context of generalized twisted drawings. We obtain the following sufficient condition for a drawing to be generalized twisted, which we also use to prove Theorem 1.1.

### Theorem 1.6

Let *D* be a simple drawing of a complete graph containing a subdrawing $D_{2,n}$, which is a plane drawing of $K_{2,n}$. Let $A=\{a_1,a_2, \ldots , a_n\}$ and $B=\{b_1,b_2\}$ be the sides of the bipartition of $D_{2,n}$. Let $D_A$ be the subdrawing of *D* induced by the vertices of *A*. Then $D_A$ is strongly isomorphic to a c-monotone drawing. Moreover, if all edges in $D_A$ cross the edge $b_1b_2$, then $D_A$ is strongly isomorphic to a generalized twisted drawing.

A weaker version of the first part of Theorem 1.6 has been implicitly shown in [15, 17], namely, the statement that in a drawing containing a plane $K_{2,n}$ as above, the subdrawing $D_A$ is weakly isomorphic to a c-monotone drawing. However, the reasoning in [15, 17] does not yield strongly isomorphic drawings. To prove Theorem 1.6, we provide an explicit construction of a strongly isomorphic c-monotone drawing, which we show to be generalized twisted if $b_1b_2$ is crossed by all edges.

The strong isomorphism in Theorem 1.6 is obtained from a result on c-monotone drawings, which we believe to be of independent interest as well. In that context, we look at quasi-monotone drawings. A drawing *D* is *quasi-x-monotone* [17] if each one of the vertical lines passing through the vertices of *D*, crosses each edge of *D* at most once (while vertical lines that do not pass through a vertex are allowed to intersect edges more than once). Similarly, the drawing *D* is *quasi-c-monotone*, if there is a point *O* such that each halfline emanating from *O* and passing through a vertex of *D* crosses each edge of *D* at most once. As pointed out by Fulek and Ruiz-Vargas [17], any quasi-x-monotone (quasi-c-monotone) drawing is weakly isomorphic to an x-monotone (c-monotone) drawing. We strengthen this result and show that any quasi-x-monotone (quasi-c-monotone) drawing is also strongly isomorphic to an x-monotone drawing (c-monotone drawing).

### Theorem 1.7

Let *D* be a quasi-x-monotone or quasi-c-monotone drawing of a graph $G=(V,E)$. Then *D* is strongly isomorphic to an x-monotone or a c-monotone drawing $\overline{D}$ of *G*, respectively.

**Outline.** In Sect. 2, we introduce some further definitions, give some preliminaries on plane drawings, and prove some useful properties of generalized twisted drawings (including Theorem 1.5). In Sect. 3, we turn to c-monotone drawings and show that quasi-c-monotone drawings are strongly isomorphic to c-monotone drawings (Theorem 1.7). In Sect. 4, we use that result to give a sufficient condition for drawings to be strongly isomorphic to generalized twisted drawings (Theorem 1.6). Sections 5 and 6 are devoted to improving the lower bound on the number of disjoint edges in any simple drawing of $K_n$ (Theorem 1.1) and the lower bound on the size of the largest plane cycle and plane path in any such drawing (Theorem 1.2), respectively. We conclude with some open questions and a short discussion on generalized twisted drawings in Sect. 7.

## Preliminaries and Further Previous Work

Before we begin with proving statements, we need to introduce some more terminology. The edges and vertices of a drawing partition the plane (or, more exactly, the plane minus the drawing) into regions, which are called the *cells* of the drawing. If a simple drawing is plane, then its cells are also called *faces*. A plane subdrawing *H* of *D* is *maximal* if every edge of $D {\setminus } H$ crosses an edge in *H*. In particular, *H* would not be plane when adding any further edge from *D*. A graph or drawing is *biconnected* if after removing any vertex it is still connected. A drawing is *outerplane* if it is plane and all vertices lie on the unbounded face of the drawing. A graph is *outerplanar* if admits an outerplane drawing. Outerplanar graphs have a smaller upper bound on their number of edges than planar graphs.

### Previous Plane Work

As mentioned before, there has been plenty of research on plane subdrawings of simple drawings. In this section, we state some further results that we will later use in our proofs. One concept we work with regularly is the one of regions that are bounded by a plane cycle. Any such plane cycle forms a Jordan curve and thus divides the plane into two connected components (the interior and the exterior, which are separated from each other via the curve) [23, 35].

Further, we will repeatedly work with maximal plane subdrawings and use the following result by García et al. [18].

#### Theorem 2.1

([18]) For $n\ge 3$, every maximal plane subdrawing of any simple drawing of $K_n$ is spanning and biconnected.

For plane subdrawings of simple drawings, Ruiz-Vargas [32, Corollary 5] showed the following. Let *D* be a simple drawing of an arbitrary graph and let *H* be a connected plane subdrawing of *D* that contains at least two vertices. Let *v* be a vertex in *D* that is not in *H* and let *F* be the face of *H* that contains *v*. Further, assume that for every vertex *w* incident to *F*, the edge *vw* is an edge of *D*. Then *D* contains two edges incident to *v* that connect *v* with vertices on the boundary of *F* such that these edges lie completely inside the face *F*. We will continuosly use the following theorem that is a reformulation of this result for the special case of simple drawings of complete graphs.

#### Theorem 2.2

([32]) Let *D* be a simple drawing of $K_n$ with $n\ge 3$. Let *H* be a connected plane subdrawing of *D* containing at least two vertices, and let *v* be a vertex in $D \setminus H$. Then *D* contains two edges incident to *v* that connect *v* with *H* and do not cross any edges of *H*.

### Twisted Preliminaries

In addition to the properties of plane (sub-)drawings from the last section, we also use several properties of generalized twisted drawings. In this section, we show most of the properties that will be used in the following sections. We start with a lemma concerning crossing properties of generalized twisted drawings.

#### Lemma 2.3

Let *D* be a generalized twisted drawing of $K_4$, with vertices $\{v_1, v_2, v_3, v_4\}$ labeled counterclockwise around *O*. Then the edges $v_1v_3$ and $v_2v_4$ do not cross.

**Fig. 3:**
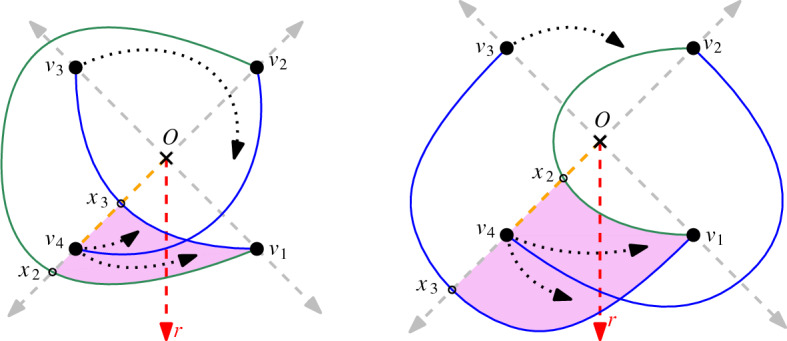
The two possibilities to draw $v_1v_3$ and $v_2v_4$ such that they cross and the drawing is generalized twisted

#### Proof

Assume, for a contradiction, that the edge $v_2v_4$ crosses the edge $v_1v_3$. Note that any simple drawing of $K_4$ has at most one crossing (a fact which can be seen by an easy case distinction). Hence all edges in *D* except $v_2v_4$ and $v_1v_3$ are uncrossed. Further, recall that in any generalized twisted drawing, all edges are drawn c-monotone and intersect the ray *r*. For every edge, this determines in which direction it emanates from its vertices. Hence, there are (up to strong isomorphism) two possibilities how the crossing edges $v_1v_3$ and $v_2v_4$ can be drawn in *D*, depending on whether $v_1v_3$ crosses the ray from *O* through $v_4$ at a point $x_3$ before or after $v_4$; see Fig. 3. In both cases, $v_1v_2$ has to cross the ray from *O* through $v_4$ at a point $x_2$. This point $x_2$ has to lie after $v_4$ in the first case and before $v_4$ in the second case. In both cases, as the edge $v_3v_4$ has to cross *r*, it must emanate from $v_4$ in the interior of the triangular region bounded by the segment $x_2x_3$, the portion $v_1x_3$ of $v_1v_3$, and the portion $v_1x_2$ of $v_1v_2$. However, the vertex $v_3$ is in the exterior of that triangular region, and therefore $v_3v_4$ would have to either cross the segment $x_2x_3$, contradicting that *D* is c-monotone, or cross $v_1v_3$, contradicting the simplicity of *D*, or cross $v_1v_2$ twice, again contradicting the simplicity of *D*. $\square$

Using the crossing property of Lemma 2.3, we next show that generalized twisted drawings always contain plane Hamiltonian paths.

#### Theorem 1.5

Every generalized twisted drawing *D* of $K_n$ contains a plane Hamiltonian path. Moreover, if $n\ge 3$ is odd, then *D* also contains a plane Hamiltonian cycle.

#### Proof

Let *D* be a generalized twisted drawing of $K_n$, with vertices $\{v_1, v_2, \ldots , v_n\}$ labeled counterclockwise around *O*. Depending on the parity of *n*, we first consider an explicit Hamiltonian path and show that it is in fact plane. Consider the Hamiltonian path $v_1, v_{\lceil \frac{n}{2}\rceil +1}, v_2, v_{\lceil \frac{n}{2}\rceil +2}, v_3, \ldots , v_{\lceil \frac{n}{2}\rceil -1}, v_n, v_{\lceil \frac{n}{2}\rceil }$ if $n \ge 3$ is odd, or the Hamiltonian path $v_1, v_{\lceil \frac{n}{2}\rceil +1}, v_2, v_{\lceil \frac{n}{2}\rceil +2}, v_3, \ldots , v_{n-1}, v_{\lceil \frac{n}{2}\rceil }, v_n$ if *n* is even. See for example the Hamiltonian path $v_1, v_4, v_2, v_5, v_3$ in Fig. 1a. Take any pair of edges $v_iv_j$ and $v_{k}v_{l}$ of the path, where we can assume without loss of generality that $i < j$ and $k < l$. If the two edges share an endpoint, they are adjacent and hence do not cross. If they do not share an endpoint, then either $i< k< j < l$ or $k< i< l < j$ by the definition of the path. In each of the two cases, $v_iv_j$ and $v_{k}v_{l}$ cannot cross by Lemma 2.3. Therefore, no pair of edges cross, implying that the Hamiltonian path is plane.

If $n \ge 3$ is odd, then this Hamiltonian path can be closed to a plane Hamiltonian cycle. The edge $v_1v_{\lceil \frac{n}{2}\rceil }$ is adjacent to $v_1v_{\lceil \frac{n}{2}\rceil +1}$. For any other edge $v_kv_l$ of the path, it holds that $1< k< \lceil \frac{n}{2}\rceil < l$. Thus, $v_1, v_{\lceil \frac{n}{2}\rceil +1}, v_2, v_{\lceil \frac{n}{2}\rceil +2}, \ldots v_{\lceil \frac{n}{2}\rceil }, v_{\lceil \frac{n}{2}\rceil -1}, v_n, v_{\lceil \frac{n}{2}\rceil }, v_1$ is plane.$\square$

We strongly conjecture that every generalized twisted drawing of $K_n$ contains a plane Hamiltonian cycle, but for even *n* this is still an open problem.

## Quasi-monotonicity and Monotonicity

The aim of this section is to prove Theorem 1.7. We will then use it to show Theorem 1.6 that we in turn will use to improve the lower bound on the number of disjoint edges.

Theorem 1.7 is a structural result on x-monotone and c-monotone drawings that we believe to be of independent interest. Specifically, it states that every quasi-x-monotone drawing is strongly isomorphic to an x-monotone drawing and every quasi-c-monotone drawing is strongly isomorphic to a c-monotone drawing. A similar result for pseudoline arrangements has been shown in [20], namely, that every arrangement of pseudolines is isomorphic to a wiring diagram.

The proofs for the x-monotone case and the c-monotone case are very similar. For better readability of the figures, we first present the proof for x-monotone drawings and then conclude with a short explanation of how the proof for c-monotone drawings is obtained.

We prove Theorem 1.7 for monotone drawings by showing how to obtain, from any quasi-x-monotone drawing *D*, a new drawing $\overline{D}$ that is strongly isomorphic to *D* and x-monotone. Moreover, each edge of $\overline{D}$ will be a polygonal chain, with vertices (turning points) only in the crossing points of $\overline{D}$ or in the crossing points of $\overline{D}$ with the vertical lines passing through the vertices of *D*.

### Procedure for Obtaining x-Monotone Drawings

**Fig. 4:**
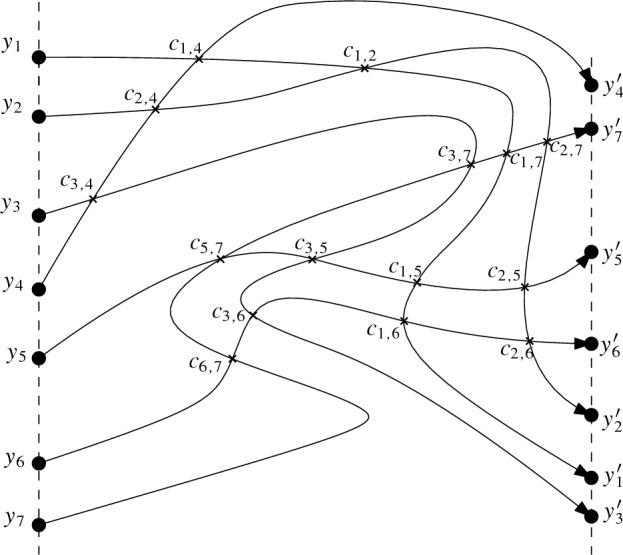
The simple drawing $D_{h,h'}$ with directed edges

Let *D* be a quasi-x-monotone drawing. We construct a monotone drawing strongly isomorphic to *D* by looking at the parts of *D* between two vertical lines separately. To that end, we describe the part of the drawing between two vertical lines *h* and $h'$ with *k* simple curves between them via a drawing $D_{h,h'}$. To define that drawing, let $r_1,\ldots ,r_k$ be the *k* simple curves that are formed by the intersection of the edges of *D* with the strip bounded by the vertical lines *h* and $h'$ through two vertices of *D* that are consecutive in x-direction. We first observe the following: (1) Any two curves $r_i$ have at most one common point (because they are parts of edges of the simple drawing *D*) and the crossings are proper crossings. (2) Each edge of *D* induces at most one $r_i$ and each curve $r_i$ connects a point $y_i$ on *h* with a point $y'_i$ on $h'$ (since *D* is quasi-x-monotone and the strip contains no vertices of *D* in its interior). Thus, taking the points $y_i$ and $y'_i$ (on *h* and $h'$) as vertices and the curves $r_i$ between them as edges, we obtain a simple drawing. We define $D_{h,h'}$ as the simple drawing obtained by this process. We suppose that the points $y_1,y_2,\ldots ,y_k$ are placed on *h* in this order (with decreasing *y*-coordinates). Some of these vertices (or even all of them) can coincide, that is, $y_i$ can coincide with $y_j$ or $y'_i$ can coincide with $y'_j$. If $r_i$ and $r_j$, with $i<j$, cross each other, we denote the corresponding crossing point by $c_{i,j}$. We direct each $r_i$ from $y_i$ to $y'_i$; see Fig. 4 for an example.

Let $D^{*}_{h,h'}$ be the planarization of $D_{h,h'}$, obtained by adding auxiliary vertices at the crossing points of $D_{h,h'}$ and subdividing the edges of $D_{h,h'}$ at those points (each crossing in $D_{h,h'}$ generates a vertex of degree four in $D^{*}_{h,h'}$). We direct each edge of $D^{*}_{h,h'}$ according to the direction of the corresponding edge $r_i$ in $D_{h,h'}$ it lies on. This way, $D^{*}_{h,h'}$ is a plane (and hence also simple) drawing of a directed planar graph. With a slight abuse of notation, we use the same label for vertices in $D^{*}_{h,h'}$ as we do for their corresponding point in $D_{h,h'}$. In particular, we use $c_{i,j}$ to refer to crossing points in $D_{h,h'}$ and vertices in $D^{*}_{h,h'}$ (which represent these crossing points). In $D^{*}_{h,h'}$, the vertices $y_i$ have indegree 0, the vertices $c_{i,j}$ have indegree 2 and outdegree 2, and the vertices $y'_i$ have outdegree 0. Any $r_i$ of $D_{h,h'}$ forms a directed path in $D^{*}_{h,h'}$ (which is a sequence of directed edges from $y_i$ to $y'_i$ connecting vertices that correspond to crossing points of $r_i$ in the same order as they occur on $r_i$ in $D_{h,h'}$).

We consider the following lemma the key ingredient of our proof.

#### Lemma 3.1

The plane directed drawing $D^{*}_{h,h'}$ is a drawing of an acyclic directed graph.

#### Proof

By definition, $D^{*}_{h,h'}$ is a plane drawing of a directed graph. To see that it is acyclic, assume for a contradiction that $D^{*}_{h,h'}$ contains some directed cycle. Let *C* be a directed simple cycle that does not contain any other such cycle in the region bounded by *C*.

We first show that *C* must consist of the edges of a face of $D^{*}_{h,h'}$ or, equivalently, there are no edges inside the region bounded by *C*.

**Fig. 5:**
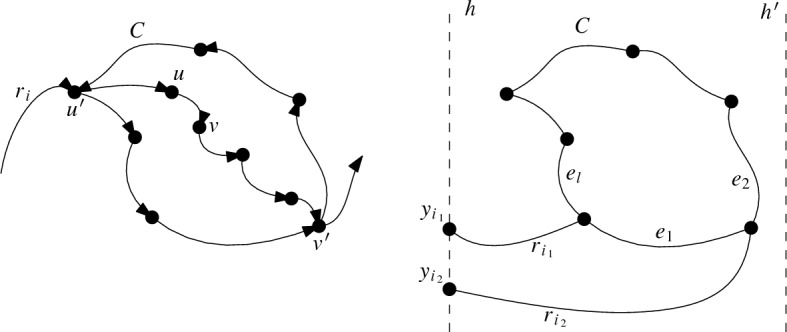
Cycle *C* of the proof of Lemma 3.1

Assume, for a contradiction, that there is an edge *uv* (directed from *u* to *v*) that lies inside the region that *C* bounds. Then *uv* belongs to a curve $r_i$ that crosses *C* in two vertices $u'$ and $v'$ and forms a directed path from $u'$ to $v'$ in $D^{*}_{h,h'}$; see Fig. 5 (left). Hence, the directed cycle formed by the path from $u'$ to $v'$ on $r_i$ and the path from $v'$ to $u'$ on *C* is contained inside the region bounded by *C*.

This contradicts the assumption that there is no directed cycle contained in the region bounded by *C*.

Now let $e_1,e_2,\ldots ,e_\ell$, $\ell \ge 3$, be the directed edges of *C* enclosing a face of $D^{*}_{h,h'}$ in this order. Suppose without loss of generality that *C* is directed counterclockwise (the reasoning for clockwise is analogous). By the construction of $D^{*}_{h,h'}$, each edge $e_j$ of *C* lies on a curve $r_{i_j}$, which is an edge in $D_{h,h'}$ starting from a vertex $y_{i_j}$ on *h* (and ending at a point $y'_{i_j}$ on $h'$). As $D_{h,h'}$ is a simple drawing, any two such curves $r_{i_j}$ can intersect at most once. For every $1 \le j \le \ell -1$, the edges $e_j$ and $e_{j+1}$ share a point on *C*. Since the vertices $y_{i_j}$ have indegree 0 and the vertices $y'_{i_j}$ have outdegree 0, they cannot be vertices of any directed cycle. Thus, each vertex of *C* has to be a crossing vertex (which is the unique intersection point of the two curves). Further, since *C* is directed counterclockwise, the starting point $y_{i_j}$ of $r_{i_j}$ must lie strictly above the starting point $y_{i_{j+1}}$ of $r_{i_{j+1}}$. Finally, since *C* is a cycle, all of these properties hold cyclically along *C*, implying that also $y_{i_{\ell }}$ lies strictly above $y_{i_1}$. Altogether, it follows that $y_{i_1}$ lies strictly below $y_{i_{\ell }}$, which in turn lies strictly below $y_{i_1}$, a contradiction. $\square$

Since $D^{*}_{h,h'}$ is a directed acyclic graph, there is a linear order $\prec$ of its vertices such that for every directed edge *uv* from *u* to *v*, vertex *u* comes before *v* in the order $\prec$. By construction, if a vertex $c_{i,j}$ is before a vertex $c_{i,j'}$ on $r_i$, then $c_{i,j}$ is before $c_{i,j'}$ in the order $\prec$. For example, in Fig. 4, the curve $r_1$ implies $c_{1,4}\!\prec \!c_{1,2}\!\prec \!c_{1,7}\!\prec \!c_{1,5}\!\prec \!c_{1,6}$. Likewise, $r_2$ implies $c_{2,4}\!\prec \!c_{1,2}\!\prec \!c_{2,7}\!\prec \!c_{2,5}\!\prec \!c_{2,6}$, and so on. Thus, a valid order for the vertices $c_{i,j}$ is $c_{3,4}\!\prec \!c_{6,7}\!\prec \!c_{5,7}\!\prec \!c_{2,4}\!\prec \!c_{1,4}\!\prec \!c_{3,7}\!\prec \!c_{3,5}\!\prec \!c_{3,6}\!\prec \!c_{1,2}\!\prec \!c_{1,7}\!\prec \!c_{1,5}\!\prec \!c_{1,6}\!\prec \!c_{2,7}\!\prec \!c_{2,5}\!\prec \!c_{2,6}$.

We now show how to obtain an x-monotone drawing $\overline{D_{h,h'}}$ that is strongly isomorphic to $D_{h,h'}$. First, we add two auxiliary vertices $y_0$ and $y_{k+1}$ to $D^{*}_{h,h'}$, both on the line *h*, $y_0$ above $y_1$, and $y_{k+1}$ below $y_k$. In the same way, we add a top point $y'_0$ and a bottom point $y'_{k+1}$ on the line $h'$. To the extended drawing, we further add the straight-line segments $y_iy_{i+1}, y'_iy'_{i+1}$ (if the endpoints are different) for $i=0,\ldots ,k$, and two more edges: $y_0y'_0$ on the top of the drawing, and $y_{k+1}y'_{k+1}$ on the bottom, both not crossing any other edge of the drawing. Denote by $D_0$ the drawing of the subgraph formed by these $\le 2(k+2)$ added edges. It forms a plane cycle with vertices on the different points $y_i,y'_i$ for $i=0,\ldots ,k+1$.

We denote the drawing of the graph consisting of $D_0$ and the first *i* curves $r_1, r_2, \ldots , r_i$ by $D_i$. In other words, $D_i$ is obtained from $D_{i-1}$ by adding $r_i$ to $D_{i-1}$. Analogously, we consider the following plane directed drawing $D^{*}_i$, which corresponds to $D_i$: The initial drawing $D^{*}_0$ is identical to $D_0$.

We will obtain piecewise straight-line (and monotone) drawings strongly isomorphic to $D^{*}_i$ and call these drawings $\overline{D^{*}_i}$. For every $\overline{D^{*}_i}$, the following invariants hold.

(i) $\overline{D^{*}_i}$ is a drawing strongly isomorphic to $D^{*}_i$.
(ii) The edges of $D^{*}_i$ on the curve $r_i$ form an x-monotone polygonal path in $\overline{D^{*}_i}$.
(iii) All the internal faces of $\overline{D^{*}_i}$ are convex.

To build $\overline{D^{*}_0}$, we keep the vertices and edges of $D^{*}_0$ except for the edges $y_0y'_0, y_{k+1}y'_{k+1}$ that are drawn straight-line. The drawing $\overline{D^{*}_0}$ vacuously satisfies Invariants (i), (ii), and (iii).

**Fig. 6:**
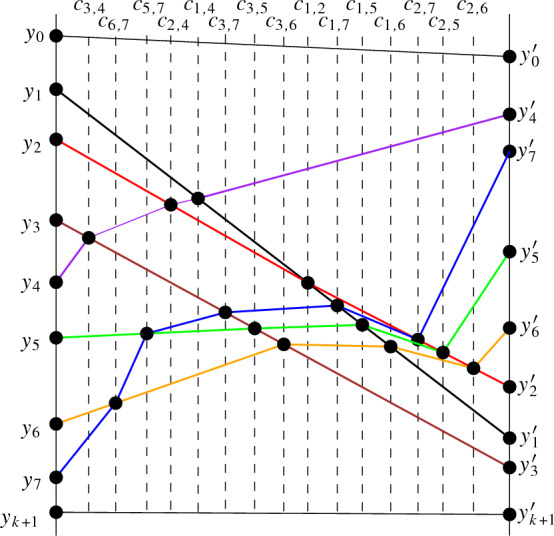
An x-monotone drawing that is strongly isomorphic to $D^{*}_{h,h'}$, with crossing vertices on the added auxiliary vertical lines

To help the remainder of the construction, we add auxiliary vertical lines to $\overline{D^{*}_0}$ which are not part of the drawing but will be used to place the crossing vertices. For each crossing vertex $c_{i,j}$ of $D^{*}_{h,h'}$, we add an auxiliary vertical line to $\overline{D^{*}_0}$ and label that line with the crossing vertex $c_{i,j}$ it will be used for. The x-order of the labeled lines is the same as the linear order $\prec$ (as described above) such that when we put each crossing vertex $c_{i,j}$ on the vertical line with that label in the following steps, the x-order of the crossing vertices will be according to the linear order $\prec$. See Fig. 6 for the final construction including the auxiliary lines.

To obtain $\overline{D^{*}_1}$ from $\overline{D^{*}_0}$, we connect $y_1$ to $y'_1$ with a straight-line segment $\overline{y_1y'_1}$. Then, if the crossings on $r_1$ are $c_{1,j_1},c_{1,j_2},\ldots$, at every intersection of the segment $\overline{y_1y'_1}$ with an auxiliary line with one of those labels $c_{1,j_1},c_{1,j_2},\ldots$, we place the corresponding crossing vertex. The subdivided segment then gives the edges for $r_1$ in $\overline{D^{*}_1}$. Observe that we obtain two convex faces $\overline{F_1},\overline{F_2}$ in $\overline{D^{*}_1}$, with the same vertices (and in the same order), edges and adjacencies as in the two faces $F_1,F_2$ of $D^{*}_1$. Thus $\overline{D^{*}_1}$ satisfies Invariants (i), (ii), and (iii).

To obtain $D^{*}_i$ from $D^{*}_{i-1}$, let $r^{*}_i$ be the path of $D^{*}_{h,h'}$ that corresponds to $r_i$. We add $r^{*}_i$ to $D^{*}_{i-1}$ (with the exact shape it has in $D^{*}_{h,h'}$). Note that $r^{*}_i$ might go through several faces of $D^{*}_{i-1}$. Further, $r^{*}_i$ enters and leaves each such face *F* of $D^{*}_{i-1}$ through some vertex of both $D^{*}_{i-1}$ and $r^{*}_i$, and splits *F* into two faces $F_1,F_2$ of $D^{*}_{i}$ (via a sub-path of $r^{*}_i$).

To obtain $\overline{D^{*}_{i}}$ from $\overline{D^{*}_{i-1}}$, we process the faces through which $r^{*}_i$ goes independently. Let *F* be a face of $D^{*}_{i-1}$ through which $r^{*}_i$ goes and let $\overline{F}$ be the corresponding face in $\overline{D^{*}_{i-1}}$. Further, let $r^{*}_{i_F}$ be the subpath of $r^{*}_i$ in *F* and let $v_F$ and $w_F$ be the endvertices of $r^{*}_{i_F}$. Note that $v_F, w_F \in \{y_i, y'_i, c_{j,i}: j<i \}$, all interior vertices of the subpath $r^{*}_{i_F}$ are in $\{c_{i,j}: j>i \}$.

Thus there are vertices $\overline{v_F}$ and $\overline{w_F}$ on the boundary of ${\overline{F}}$, which correspond to $v_F$ and $w_F$, respectively. Further, the vertices of $r^{*}_{i_F}$ are ordered along $r^{*}_{i_F}$ according to the linear order $\prec$. Hence ${\overline{F}}$ is intersected by an auxiliary vertical line labeled $c_F$ for every interior vertex $c_F$ of the subpath $r^{*}_{i_F}$. Moreover, all those lines lie between the vertical lines through $\overline{v_F}$ and $\overline{w_F}$, in the order of the vertices on $r^{*}_{i_F}$. We draw the subpath $\overline{r^{*}_{i_F}}$ for $r^{*}_{i_F}$ in ${\overline{F}}$ along the straight-line segment with endpoints $\overline{v_F}$ and $\overline{w_F}$. We place its vertices on all vertical lines that correspond to vertices of $r^{*}_{i_F}$.

Observe that, as all the vertices of $r^{*}_{i}$ obey the linear order $\prec$, the resulting polygonal path $\overline{r^{*}_{i}}$ is monotone (both within each face and in total) and Invariants (i) and (ii) are satisfied. Further, as $\overline{r^{*}_{i_F}}$ goes along a straight-line segment, the resulting faces $\overline{F_1},\overline{F_2}$ in $\overline{D^{*}_i}$ are convex, so Invariant (iii) is satisfied as well.

Performing the construction for all of $r_1,\ldots ,r_k$, we obtain a straight-line drawing $\overline{D^{*}_k}$, which yields an x-monotone drawing $\overline{D_k}\!=\!\overline{D_{h,h'}}$ that is strongly isomorphic to $D_{h,h'}$.

### Proof of Theorem 1.7

With the procedure from the previous section, we are now ready to prove Theorem 1.7.

#### Theorem 1.7

Let *D* be a quasi-x-monotone or quasi-c-monotone drawing of a graph $G=(V,E)$. Then *D* is strongly isomorphic to an x-monotone or a c-monotone drawing $\overline{D}$ of *G*, respectively.

#### Proof

We first prove the statement for quasi-x-monotone drawings. We draw all the vertical lines passing through the vertices of *D*, thus partitioning *D* into drawing parts $D_{h_1,h_2},D_{h_2,h_3},\ldots , D_{h_{n-1},h_{n}}$, where each of them is the part of *D* between two consecutive vertical lines (like the graph $D_{h,h'}$ described in Sect. 3.1).

Using the above procedure, we obtain strongly isomorphic rectilinear drawing parts $\overline{D_{h_1,h_2}},\overline{D_{h_2,h_3}},\ldots , \overline{D_{h_{n-1},h_n}}$ whose union forms an x-monotone drawing $\overline{D}$ of *G* strongly isomorphic to *D*. (Strictly speaking, the strong isomorphism for the drawing parts requires auxiliary vertices and edges to turn each drawing part into a drawing. These are disregarded for the drawing $\overline{D}$ of *G*.)

It remains to show the statement for quasi-c-monotone drawings. The method to show the statement is essentially the same as the one for quasi-x-monotone drawings. However, instead of having the parts $D_{h,h'}$ being strips in the quasi-x-monotone drawing, the parts in the quasi-c-monotone drawing are wedges with *O*, and rays *h* and $h'$ on the boundary. To avoid difficulties with straight-line segments in a wedge that is not convex, the quasi-c-monotone drawing first gets transformed by radially stretching the wedges such that all wedges are convex. (This process is an isotopy, so the resulting drawing is strongly isomorphic to the original one.) Then, the procedure for quasi-x-monotone drawings is applied to the (transformed) quasi-c-monotone drawing but we replace “vertical lines” with rays through *O*. That way each $D_{h,h'}$ (between consecutive rays *h* and $h'$) is a convex wedge and all connections within a wedge stay inside the wedge when applying the procedure. Thus, we obtain a c-monotone drawing strongly isomorphic to the original quasi-c-monotone drawing.$\square$

## Possible Extension Containing a Plane $K_{2,n}$ Implies Strong Isomorphism

### Theorem 1.6

Let *D* be a simple drawing of a complete graph containing a subdrawing $D_{2,n}$, which is a plane drawing of $K_{2,n}$. Let $A=\{a_1,a_2, \ldots , a_n\}$ and $B=\{b_1,b_2\}$ be the sides of the bipartition of $D_{2,n}$. Let $D_A$ be the subdrawing of *D* *induced by the vertices of* *A*. Then $D_A$ is strongly isomorphic to a c-monotone drawing. Moreover, if all edges in $D_A$ cross the edge $b_1b_2$, then $D_A$ is strongly isomorphic to a generalized twisted drawing.

As explained before, Theorem 1.6 is a stronger version of a lemma implicitly shown in [15, 17]. We remark that the proof presented here is in parts similar to the one in [17].

### Proof

We call the pair of edges in $D_{2,n}$ incident to $a_i$, $1 \le i \le n$, the long edge $r_i$. Let $R_{A}$ be the set of long edges. We first show that any edge between vertices in *A* crosses any long edge at most once. Then we will use $D_{2,n}$ to obtain a simple drawing which is essentially strongly isomorphic to *D*, but with $b_2$ at infinity. In this drawing, all long edges as well as $b_1b_2$ are infinite rays starting from $b_1$, and the subdrawing corresponding to $D_A$ is c-monotone with origin $b_1$.

We now show that every edge between two vertices of *A* crosses every long edge of $R_A$ at most once. Let $a_1$, $a_2$, and $a_3$ be vertices in *A*. Let $R_1$ be the region bounded by the edges $b_1a_1$, $a_1b_2$, $b_2a_2$ and $a_2b_1$ that does not contain $a_3$. Let $R_2$ be the region bounded by the edges $b_1a_2$, $a_2b_2$, $b_2a_3$ and $a_3b_1$ that does not contain $a_1$. Since $D_{2,n}$ is plane, these regions are disjoint.

As the edge $e=a_1a_2$ is incident to all edges on the boundary of $R_1$, it cannot cross it. Thus, *e* has to lie either completely inside or completely outside $R_1$ (and meet the boundary only in its endvertices). If *e* lies inside $R_1$, it can cross neither $a_3b_1$ nor $a_3b_2$. If it lies outside $R_1$, it has to cross the boundary of $R_2$ an odd number of times. (Since *e* must begin at $a_1$ outside $R_2$ and finish at $a_2$ inside $R_2$, and passing through $R_1$ is not possible.) As *e* cannot cross edges incident to $a_2$, this means it has to cross exactly one of the edges $a_3b_1$ or $a_3b_2$. Thus, *e* crosses the long edge $r_3$ at most once, for any vertex $a_3$.

**Fig. 7:**
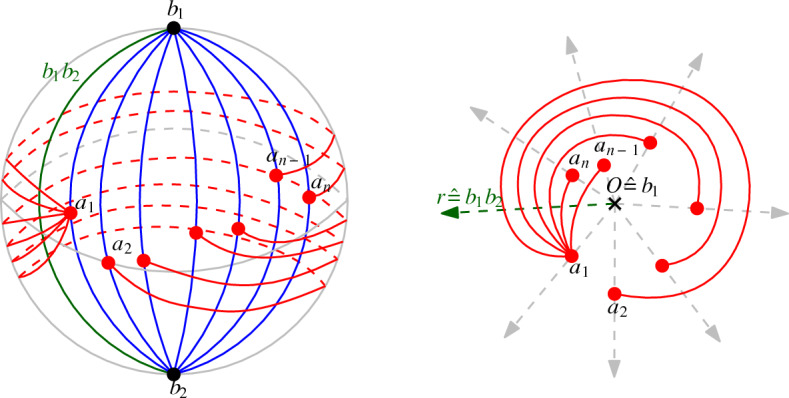
The homeomorphisms of $D_{2,n}$. Left: $D_A$, the edges in $R_{A}$ and $b_1b_2$ are drawn on the sphere, such that $R_{A}$ and $b_1b_2$ are meridians. Right: The stereographic projection from $b_2$

We now transform *D* to obtain a drawing in which $b_2$ is at infinity, all long edges as well as $b_1b_2$ are infinite rays starting from $b_1$, and the subdrawing induced by the vertices of *A* is strongly isomorphic to $D_A$ and c-monotone with origin $b_1$ in the following way; see Fig. 7. We first draw *D* on the sphere such that $b_1$ and $b_2$ are antipodes and the long edges of $R_{A}$ as well as the edge $b_1b_2$ are meridians. By the general Jordan–Schoenflies theorem [11, 25], this can be done in a way that the drawing on the sphere is homeomorphic to the original drawing *D* in the plane. We then apply a stereographic projection from $b_2$ onto the plane. This way, the long edges in $R_{A}$ and the edge $b_1b_2$ are projected to rays emerging from vertex $b_1$, where the long edges in $R_{A}$ are exactly the rays through the vertices of $D_{2,n}$. The resulting drawing after these transformations is still strongly isomorphic to *D* (as all we did was apply a stereographic projection from a homeomorphic drawing), except that $b_2$ is at infinity.

Let $D'_A$ be the subdrawing corresponding to $D_A$ after the stereographic projection. As all edges of $D_A$ cross the long edges in $R_{A}$ only once, $D'_A$ is quasi-c-monotone and thus, by Theorem 1.7, strongly isomorphic to a c-monotone drawing.

Finally, if all edges of $D_A$ cross the edge $b_1b_2$, then all edges in the quasi-c-monotone drawing $D'_A$ cross the ray $r'$ corresponding to $b_1b_2$. Let *W* be the wedge that contains $r'$, is bounded by two rays emanating from *O* and passing through vertices of $D'_A$, and does not contain any vertices of $D'_A$ in its interior. Since all edges of $D'_A$ cross $r'$, they all have to lie at least partially in *W*. As $D'_A$ is quasi-c-monotone, each edge of $D'_A$ has to intersect each of the two rays bounding *W* exactly once (either emanating from a vertex on the ray or crossing the ray). Those two intersection points are connected via a continuous part of the edge that lies completely inside *W*. Thus, after applying Theorem 1.7 to $D'_A$ (and to the subdrawing in *W* in particular), we obtain a c-monotone drawing $\overline{D'_A}$ in which any ray in the wedge corresponding to *W* crosses all edges of $\overline{D'_A}$. Therefore, $D'_A$ is strongly isomorphic to a generalized twisted drawing.$\square$

## Disjoint Edges in Simple Drawings

In this section, we show that every simple drawing of $K_n$ contains at least $\lfloor \sqrt{\frac{n}{48}} \rfloor$ pairwise disjoint edges, improving the best previously published bound of $\Omega (n^{\frac{1}{2}-\varepsilon })$, for any $\varepsilon >0$, by Ruiz-Vargas [33].

### Theorem 1.1

Every simple drawing of $K_n$ contains at least $\lfloor \sqrt{\frac{n}{48}} \rfloor$ pairwise disjoint edges.

### Proof

Let *D* be a simple drawing of $K_n$, and let *M* be a maximal plane matching of *D*. If $m:= |M| \ge \sqrt{\frac{n}{48}}$, then Theorem 1.1 holds. So assume that $|M| < \sqrt{\frac{n}{48}}$. We will show how to find another plane matching, whose size is at least $\lfloor \sqrt{\frac{n}{48}} \rfloor$.

The overall idea is the following: Let *H* be a maximal plane subdrawing of *D* whose vertex set is exactly the vertices matched in *M* and that contains *M*. We will find a face *f* in *H* that contains much more unmatched vertices of *D* inside than matched vertices on its boundary. Then we will show that there exists a subset of the vertices inside that face, which induces a subdrawing of *D* that is strongly isomorphic to a generalized twisted drawing and contains enough vertices to guarantee the desired size of the plane matching.

To find the face *f*, we start by using that *H* is biconnected by Theorem 2.1. Thus, *H* partitions the plane into faces, where the boundary of each face is a simple cycle. Note that the vertices of *H* are exactly the vertices that are matched in *M*, and the vertices inside faces are the vertices that are unmatched in *M*.

Let *U* be the set of vertices of *D* that are not matched by any edge of *M*. We denote the set of vertices of *U* inside a face $f_i$ by $U(f_i)$, the number of vertices in $U(f_i)$ by $u(f_i)$, and the number of vertices on the boundary of the face $f_i$ by $|f_i|$.

We next show that there exists a face *f* of *H* such that $u(f) \ge {\frac{\sqrt{48n}}{12}}|f|$. Assume for a contradiction that for every face $f_i$ it holds that

$$\begin{aligned} u(f_i) < {\frac{\sqrt{48n}}{12}}|f_i|. \end{aligned}$$

There are exactly $n-2m$ unmatched vertices. As every unmatched vertex is in the interior of a face of *H* (that might be the unbounded face), we can count all unmatched vertices by summing over the number of vertices in each face (including the unbounded face). By the observation above that all matched vertices are vertices on *H* (and thus on the boundary of the faces), it follows that $n-2\,m = \sum _{f_i}u(f_i)$. By the assumption on $u(f_i)$, it follows that $\sum _{f_i}u(f_i) < {\frac{\sqrt{48n}}{12}}\sum _{f_i}|f_i|$. Consequently,

1 $$\begin{aligned} n-2m < {\frac{\sqrt{48n}}{12}}\sum _{f_i}|f_i|. \end{aligned}$$

The number of edges in *H* is $\frac{1}{2}\sum _{f_i}|f_i|$. Since *H* is plane, by the well known Euler formula, the number of edges of *H* is at most $3n'-6$, where $n'$ is the number of vertices in *H*. As the vertices of *H* are exactly the matched vertices, their number is $n'=2m$. Hence,

$$\begin{aligned}\sum _{f_i}|f_i|\le 6 \cdot 2m-12. \end{aligned}$$

From $m < \sqrt{\frac{n}{48}}$ it follows that

2 $$\begin{aligned} \sum _{f_i}|f_i| < 12\sqrt{\frac{n}{48}}-12, \end{aligned}$$

and

3 $$\begin{aligned} n-2 \sqrt{\frac{n}{48}} < n-2m. \end{aligned}$$

Putting Eqs. (1) to (3) together we obtain that

$$\begin{aligned}n-2 \sqrt{\frac{n}{48}} < {\frac{\sqrt{48n}}{12}} \left( 12\sqrt{\frac{n}{48}}-12\right) =n - \sqrt{48n}. \end{aligned}$$

However, this inequality cannot be fulfilled by any $n \ge 0$. Thus, there exists at least one face $f_i$ with $u(f_i) \ge {\frac{\sqrt{48n}}{12}}|f_i|$. We take such a face and call it *f*.

**Fig. 8:**
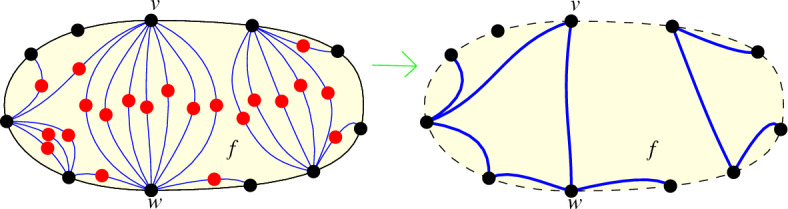
Left: The face *f* in *H* containing the plane drawing $H'$ (blue lines) inside. Right: We can obtain an outerplane drawing from $H'$ by interpreting bundles of edge pairs incident to the same black vertices as plane edges

As a next step, we will find two vertices on the boundary of *f* to which many vertices inside *f* are connected via edges that do not cross each other or *H*. From *f* and the set *U*(*f*), we construct a plane subdrawing $H'$ as follows; cf. Fig. 8 (left). We add the vertices and edges on the boundary of *f* to $H'$. Then we iteratively add all the vertices in *U*(*f*) to $H'$. By Theorem 2.2, for each vertex *v* in *U*(*f*) that is not yet in $H'$ there are at least two edges of *D* incident to *v* that can be added such that the resulting drawing stays plane. For each vertex *v* that we add, we also always add exactly two such edges to $H'$. Since the matching *M* is maximal plane, every edge between two unmatched vertices must cross at least one edge of *M* and thus must cross the boundary of *f*. Hence, no edge in $H'$ can connect two vertices of *U*(*f*) (as they are unmatched). Consequently, every vertex in *U*(*f*) is connected in $H'$ to exactly two vertices that both lie on the boundary of *f*.

We consider the two edges in $H'$ that connect a vertex in *U*(*f*) to *f* as a pair of edges. Every edge in such a pair is incident to exactly one unmatched vertex (namely, the one it is added for) and hence contained in no other pair. Thus, we can see every such pair of edges as one *long edge* incident to two vertices on the boundary of *f*. We call every group of long edges that have the same endpoints a *bundle of edges*; see Fig. 8 (right).

From the long edges, we can define a graph $G'$ as follows. The vertices of $G'$ are the vertices of *D* that lie on the boundary of *f*. Two vertices *u* and *v* are connected in $G'$ if there is at least one long edge in $H'$ that connects them. By the definition of long edges, $G'$ is outerplanar (as can be observed in Fig. 8 (right)). It is well known that the number of edges in an outerplanar graph with $n'$ vertices is at most $2n'-3$ [26]. Thus, $G'$ has at most $2|f|-3$ edges. On the other hand, every unmatched vertex in *U*(*f*) defines a long edge, so the number of long edges is $u(f) \ge \frac{\sqrt{48n}}{12}|f|$. As a consequence, there is a pair of vertices on the boundary of *f* such that the number of long edges in its bundle is at least

$$\begin{aligned} {\frac{1}{(2 |f|-3)}}\frac{\sqrt{48n}}{12}|f|> \frac{\sqrt{48n}}{{24}}. \end{aligned}$$

**Fig. 9:**
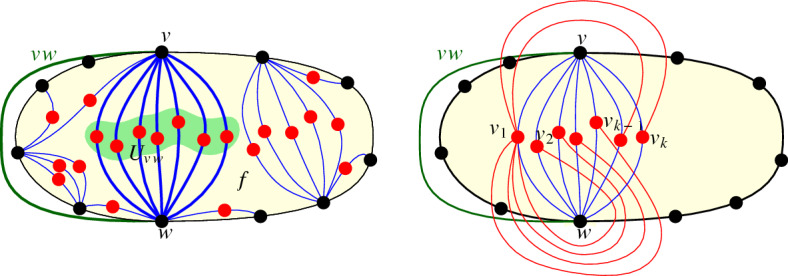
The subdrawing $D_{vw}$ induced by $U_{vw}$ and the edges in $D_{vw}$. Left: The set $U_{vw}$. Right: The edges adjacent to the leftmost vertex, $v_1$, are drawn (in red)

This implies that there are two vertices, say *v* and *w*, such that for more than $\frac{\sqrt{48n}}{{24}}$ vertices in *U*(*f*) the two plain edges connecting them to *f* are incident to *v* and *w*. We denote the set of vertices in *U*(*f*) that have plane edges to both vertices *v* and *w* by $U_{vw}$. This set is marked in Fig. 9 (left). We denote the subdrawing of *D* induced by $U_{vw}$ by $D_{vw}$; see Fig. 9 (right).

**Fig. 10:**
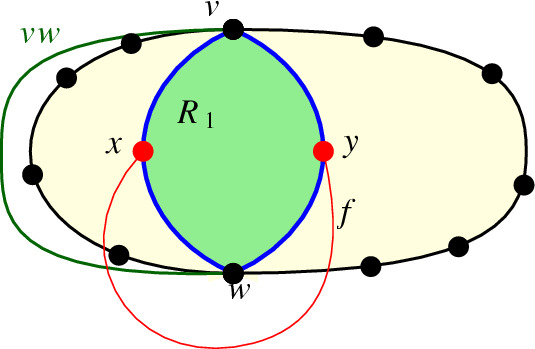
The edge *xy* has to cross the edge *vw*

We show that all edges between vertices in $U_{vw}$ cross the edge *vw*. Let *x* and *y* be two vertices of $D_{vw}$. Let $R_1$ be the region bounded by the edges *xv*, *vy*, *yw*, and *wx* that lies inside the face *f*; see Fig. 10. We show that *xy* and *vw* lie completely outside $R_1$. The edge *xy* has to lie either completely inside or completely outside $R_1$ because it is adjacent to all edges on the boundary of $R_1$. As *M* is maximal and the edge *xy* connects two unmatched vertices, it has to cross at least one matching edge. Thus, *xy* has to lie completely outside $R_1$. (There can be no matching edges in $R_1$, as $R_1$ is contained inside the face *f*.)

Note that as *H* is a maximal plane subdrawing, *vw* cannot lie completely inside the face *f*. Since both edges *vw* and *xy* lie completely outside $R_1$ and the vertices along the boundary of $R_1$ are sorted *vxwy*, the two edges have to cross. Thus, all edges of $D_{vw}$ cross the edge *vw*.

Since the edges from vertices in $U_{vw}$ to *v* and *w* are plane, it follows from Theorem 1.6 that $D_{vw}$ is strongly isomorphic to a generalized twisted drawing. Thus, $D_{vw}$ contains at least $\lfloor \frac{1}{2}\frac{\sqrt{48n}}{{24}}\rfloor$ pairwise disjoint edges by Theorem 1.5. Hence, *D* contains at least $\lfloor \sqrt{\frac{n}{48}}\rfloor$ pairwise disjoint edges.$\square$

## Plane Cycles and Paths in Simple Drawings

In the previous section, we used generalized twisted drawings to improve the lower bound on the number of disjoint edges in simple drawings of $K_n$. In this section, we show that generalized twisted drawings are also helpful to obtain a lower bound of $\Omega (\frac{\log n }{\log \log n})$ on the length of the longest plane cycle, where the length of a cycle (or path) is the number of its edges.

### Theorem 1.2

Every simple drawing of $K_n$ contains a plane cycle of length $\Omega (\frac{\log n }{\log \log n})$.

To prove the lower bound for cycles, we first show that all c-monotone drawings of $K_n$ contain either a generalized twisted drawing of $K_{\sqrt{n}}$ or a drawing strongly isomorphic to an x-monotone drawing of $K_{\sqrt{n}}$. We know that drawings weakly isomorphic to generalized twisted drawings or x-monotone drawings of complete graphs contain plane Hamiltonian cycles (by Theorem 1.5 and Observation 6.1 below). We conclude that c-monotone drawings of $K_n$ contain plane cycles of the desired size. Essentially, we then show that for any $1 \le d \le n-2$ every simple drawing of the complete graph contains either a c-monotone drawing of $K_d$, or a plane spanning tree of maximal degree at most *d* (plus more edges). With easy observations about the length of the longest path in bounded degree trees and by putting all results together, we obtain that every simple drawing *D* of $K_n$ contains a plane cycle of length $\Omega (\frac{\log n }{\log \log n})$.

### Plane Cycles in C-Monotone Drawings

**Fig. 11:**
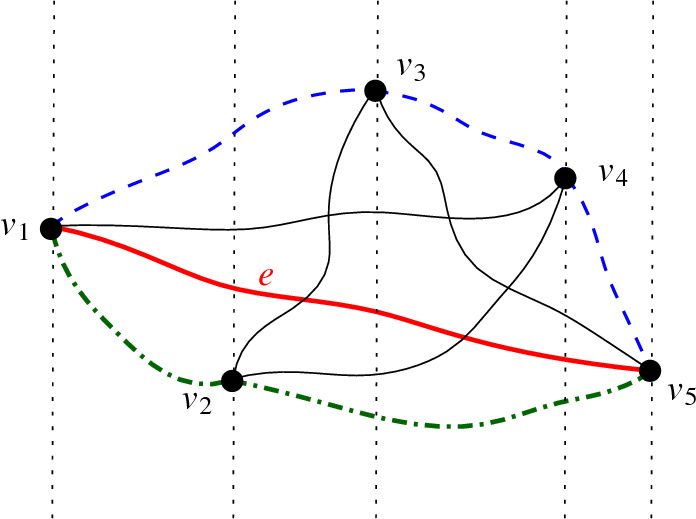
An x-monotone drawing of $K_5$ with a plane Hamiltonian cycle, consisting of the (blue, dashed) plane path above the (bold, red) edge *e* and the (green, dash-dotted) plane path below *e*

It is well-known that any x-monotone drawing *D* of $K_n$ contains a plane Hamiltonian cycle. For instance, consider the edge *e* between the vertices $v_1$ and $v_n$ with the smallest and largest, respectively, x-coordinates; see for example the bold, red edge in Fig. 11. The edge *e* splits the remaining vertices into two subsets, namely, the ones above *e* and the ones below *e*, where $v_1$ and $v_n$ can be seen as belonging to both subsets. For each subset, the vertices in x-order form a plane path in *D*, and none of those paths can cross *e*. Hence, the union of the two paths is a plane Hamiltonian cycle of *D*. Recall that every monotone drawing is strongly isomorphic to an x-monotone drawing by definition. Therefore, we have the following observation.

#### Observation 6.1

Every monotone drawing of $K_n$ contains a plane Hamiltonian cycle.

We will show that c-monotone drawings contain plane cycles of size at least $\sqrt{n}$, by showing that any c-monotone drawing of $K_n$ contains a subdrawing of $K_{\sqrt{n}}$ that is either generalized twisted or monotone. To do so, we will use Dilworth’s Theorem on chains and anti-chains in partially ordered sets. A *chain* is a subset of a partially ordered set such that any two distinct elements are comparable. An *anti-chain* is a subset of a partially ordered set such that any two distinct elements are incomparable.

#### Theorem 6.2

(Dilworth’s Theorem, [12]) Let *P* be a partially ordered set of at least $(s\!-\!1)(t\!-\!1)\!+\!1$ elements. Then *P* contains a chain of size *s* or an antichain of size *t*.

#### Theorem 6.3

Let *s*, *t* be two integers, $1\le s,t\le n$, such that $(s-1)(t-1)+1\le n$. Let *D* be a c-monotone drawing of $K_n$. Then *D* contains either a generalized twisted drawing of $K_s$ or a monotone drawing of $K_t$ as subdrawing. In particular, if $s=t= \lceil \sqrt{n} \rceil$, *D* contains a complete subgraph $K_s$ whose induced drawing is either generalized twisted or monotone.

#### Proof

Without loss of generality, we may assume that the vertices of *D* appear counterclockwise around *O* in the order $v_1,v_2,\ldots ,v_n$. Let *r* be a ray emanating from *O* in the wedge defined by *O*, $v_1$, and $v_n$, that is, *r* is such that when rotating *r* clockwise, the first vertex it encounters is $v_1$, and when rotating *r* counterclockwise, the first vertex is $v_n$.

We define an order, $\preceq$, in this set of vertices as follows: $v_i\preceq v_j$ if and only if either $i=j$ or $i<j$ and the edge $v_iv_j$ crosses *r*.

**Fig. 12:**
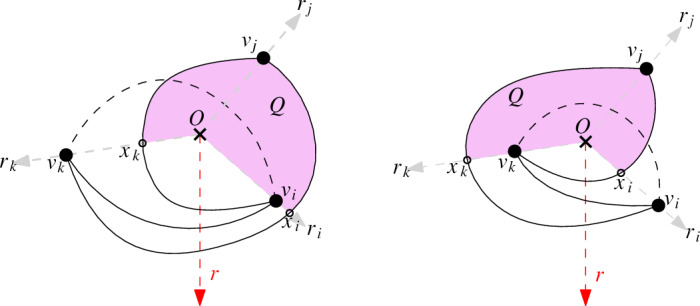
If edges $v_iv_j$ and $v_jv_k$ cross *r* in a c-monotone drawing, then $v_iv_k$ must also cross *r*

We show that $\preceq$ is a partial order. The relation is clearly reflexive and antisymmetric. Besides, if $v_i\preceq v_j$ and $v_j\preceq v_k$, then $i<j$ and $j<k$ imply $i<k$, so for the transitive property, we only have to prove that if $v_iv_j$ and $v_jv_k$ cross *r*, then $v_iv_k$ also crosses *r*. We denote the rays emanating from *O* and passing trough $v_i,v_j,v_k$ by $r_i,r_j,r_k$, respectively. We have two cases depending on where $v_jv_i$ crosses the ray $r_k$ at a point $x_k$; in the first case, $x_k$ is located before $v_k$ on $r_k$, while in the second one $x_k$ is located after $v_k$. Then $v_jv_k$ has to cross the ray $r_i$ at a point $x_i$, which is after $v_i$ in the first case and before $v_i$ in the second case; see Fig. 12. Let *Q* be the region bounded by the segments $Ox_i,Ox_k$ and the portions $v_jx_i$ and $v_jx_k$ of the edges $v_jv_k$ and $v_jv_i$, respectively. By simplicity, the edge $v_iv_k$ cannot cross the edges $v_iv_j$ and $v_jv_k$. As in both cases a vertex of $\{v_i,v_k\}$ is placed outside *Q*, the edge $v_iv_k$ thus cannot be contained in the counterclockwise wedge from $r_i$ to $r_k$ without contradicting the c-monotonicity. Therefore, $v_iv_k$ must be in the clockwise wedge from $r_i$ to $r_k$ and thus crosses the ray *r*.

In this partial order $\preceq$, a chain consists of a subset $v_{i_1},\ldots ,v_{i_{s-1}}$ of pairwise comparable vertices, that is, a subset of vertices such that their induced subdrawing is generalized twisted (all edges cross *r*). An antichain, $v_{j_1},\ldots ,v_{j_{t-1}}$, consists of a subset of pairwise incomparable vertices, that is, a subset of vertices such that their induced subdrawing is monotone (no edge crosses *r*).

Therefore, the first part of the theorem follows from applying Theorem 6.2 to the set of vertices of *D* and the partial order $\preceq$.

Finally, observe that if $s=t\le \lceil \sqrt{n}\rceil$, then $(s-1)(t-1)+1\le n$. Thus, *D* contains a complete subgraph $K_{\lceil \sqrt{n}\rceil }$ whose induced subdrawing is either generalized twisted or monotone.$\square$

Combining Theorem 6.3 with Theorem 1.5 (on $K_{\lceil \sqrt{n}\rceil }$ if $\lceil \sqrt{n}\rceil$ is odd and $K_{\lceil \sqrt{n}\rceil -1}$ otherwise) and Observation 6.1, we obtain the following theorem.

#### Theorem 6.4

Every c-monotone drawing of $K_n$ contains a plane cycle of length $\Omega (\sqrt{n})$.

### Plane Cycles in Simple Drawings

To show that any simple drawing of $K_n$ contains a plane cycle of length $\Omega (\frac{\log n }{\log \log n})$, we will use *d*-ary trees. A *d*-ary tree is a rooted tree in which no vertex has more than *d* children. It is well-known that the height (and hence also the length of the longest path) of a *d*-ary tree on *n* vertices is $\Omega (\frac{\log n}{\log d})$.

**Fig. 13:**
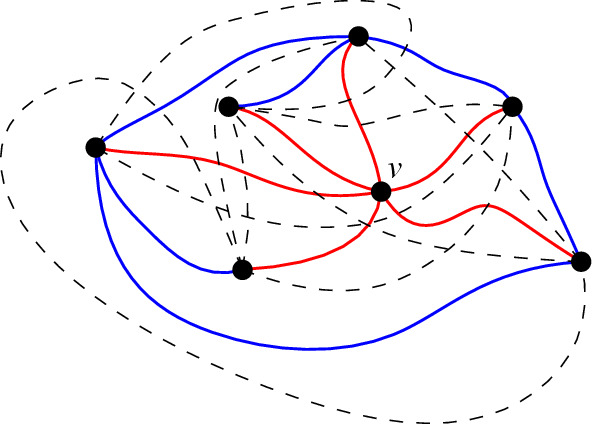
A maximal plane subdrawing *H* containing the star *S*(*v*). Dashed edges are edges of $K_7$ that are not in *H*

#### Proof of Theorem 1.2

Let *v* be a vertex of *D* and let *S*(*v*) be the star centered at *v*, that is, the set of edges of *D* incident to *v*. *S*(*v*) can be extended to a maximal plane subdrawing *H* that must be biconnected by Theorem 2.1. See Fig. 13 for a depiction of *S*(*v*) and *H*.

Assume first that there is a vertex *w* in $H\setminus v$ that has degree at least $(\log n)^2$ in *H*. Let $U_{vw}$ be the set of vertices neighbored in *H* to both, *v* and *w*. Note that $|U_{vw}| \ge (\log n)^2$. The subdrawing $H'$ of *H* consisting of the vertices in $U_{vw}$, the vertices *v* and *w*, and the edges from *v* to vertices in $U_{vw}$, and from *w* to vertices in $U_{vw}$ is a plane drawing of $K_{2,|U_{vw}|}$. From Theorem 1.6, it follows that the subdrawing of *D* induced by $U_{vw}$ is strongly isomorphic to a c-monotone drawing. Therefore, by Theorem 6.4, the subdrawing induced by $U_{vw}$ contains a plane cycle of length $\Omega (\sqrt{|U_{vw}|}) = \Omega (\log n)$.

Assume now that the maximum degree in $H\setminus v$ is less than $(\log n)^2$. Since *H* is biconnected, $H\setminus v$ contains a plane tree *T* of order $n-1$ whose maximum degree is at most $(\log n)^2$. Considering *T* as rooted (choosing an arbitrary vertex as root), it is a $(\log n)^2$-ary tree and thus has height at least $\Omega (\frac{\log n }{\log \log n})$.

Therefore, since *T* is plane, it contains a plane path of length at least $\Omega (\frac{\log n }{\log \log n})$. This plane path is edge-disjoint from the star of *v*, and the union of the path with the star of *v* is in *H* and thus plane. Hence, the union of the obtained plane path with the edges from *v* to the start- and endpoint of the path is a plane cycle and the theorem follows. $\square$

## Conclusion and Outlook

We used properties of generalized twisted drawings for investigating plane substructures in (general) simple drawings of complete graphs. We improved the lower bound on the number of disjoint edges in simple drawings of $K_n$ to $\Omega (\sqrt{n})$, and the lower bound on the length of the plane cycle (and hence path) contained in every simple drawing of $K_n$ to $\Omega (\frac{\log n }{\log \log n})$. However, resolving Rafla’s conjecture on the existence of plane Hamiltonian cycles, as well as its weaker versions for paths and matchings, remain wide open.

### Open Problem 1

Does every simple drawing of $K_n$ contain a

- plane Hamiltonian cycle (if $n\ge 3$)?
- plane Hamiltonian path?
- plane perfect matching (if *n* is even)?

For obtaining the improved bounds, we have shown several properties of generalized twisted drawings. For example, we have proven that every generalized twisted drawing on an odd number of vertices contains a plane Hamiltonian cycle. In that context, one natural open question on generalized twisted drawings and plane substructures is the following (where we strongly conjecture the answer to be to the positive).

### Open Problem 2

Does every generalized twisted drawing of $K_n$ contain a plane Hamiltonian cycle?

Generalized twisted drawings also have more structure, which is not included in this paper. For example, we have shown [19] that every generalized twisted drawing contains exactly $2n-4$ empty triangles, which is the conjectured lower bound on the number of empty triangles that any simple drawing of $K_n$ must contain. Further, in the conference version of this paper [7], we presented a characterization for drawings that are weakly isomorphic to generalized twisted drawings; see there for details. Due to suggestions by several reviewers to relevantly shorten the paper, this characterization is not included in this version. Instead, it will appear in a forthcoming work, in which we will also add the new equivalent property that drawings that are weakly isomorphic to generalized twisted drawings are also strongly isomorphic to generalized twisted drawings (the proof for strong isomorphism uses Theorem 1.6).

**Fig. 14:**
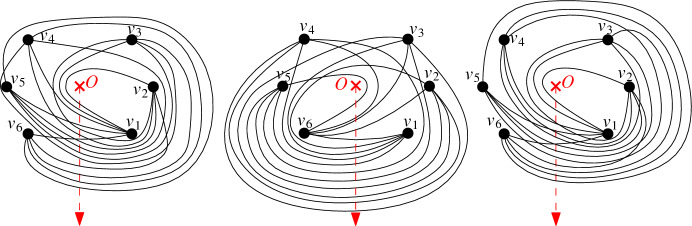
All (up to weak isomorphism) different generalized twisted drawings of $K_6$. The rightmost drawing is twisted

In the conference version [7], we used the above-mentioned characterization to obtain all generalized twisted drawings of $4 \le n \le 6$ up to weak isomorphism. The (up to strong isomorphism) only simple drawing of $K_4$ that is strongly isomorphic to a generalized twisted drawing is the drawing of $K_4$ with a crossing. Thus, generalized twisted drawings are maximal crossing. The (up to strong isomorphism) only drawing of $K_5$ that is strongly isomorphic to a generalized twisted drawing is the twisted drawing of $K_5$. This no longer holds for $n \ge 6$. For $n=6$, we computationally checked all 102 [2] weak isomorphism classes of simple drawings of $K_6$, and only three different classes have representatives that are generalized twisted; see Fig. 14 for a representation of each of the three classes. (Also, they can be extended to arbitrarily large generalized twisted drawings that are not weakly isomorphic.) Using a similar approach, we meanwhile computed the number of such weak isomorphism classes of $K_n$ for $n \le 15$ [22] and obtained the numbers shown in Table 1. This indicates that the number of generalized twisted drawings seems to be relevantly growing for increasing *n* (while there is always exactly one weak isomorphism class that has a twisted representation).

**Table 1:** Number *t*(*n*) of weak isomorphism classes of generalized twisted drawings for $5 \le n \le 15$

*n*	5	6	7	8	9	10	11	12	13	14	15
*t*(*n*)	1	3	9	32	115	443	1 723	6 875	27 663	112 629	461 734

## Footnotes

1 An extended abstract of this result has been previously presented at CG:YRF 2021 [5]. We recently learned that the PhD thesis of Ruiz-Vargas also contains a bound of $\Omega (n^{\frac{1}{2}})$, which, according to personal communication with Ruiz-Vargas, is an extension of [33]. However, the thesis is not publicly available and the extended result itself has not been published.

2 They use the terms ‘monotone simple cylindrical graphs’ and ‘cylindrical drawings’ instead of ‘c-monotone drawings’. However, the term ‘cylindrical drawings’ has also been used for other types of drawings in the literature; see for example [1] and references therein.

3 In their definition for simple drawings, *convex* means that there is a labeling of the vertices to $v_1, v_2, \ldots , v_n$ such that $v_iv_j$ ($i<j$) crosses $v_kv_l$ ($k<l$) if and only if $i<k<j<l$ or $k<i<l<j$.
